# Bacterial Diversity Associated with the Coccolithophorid Algae *Emiliania huxleyi* and *Coccolithus pelagicus* f. *braarudii*

**DOI:** 10.1155/2015/194540

**Published:** 2015-07-26

**Authors:** David H. Green, Virginia Echavarri-Bravo, Debra Brennan, Mark C. Hart

**Affiliations:** ^1^Microbial & Molecular Biology, Scottish Association for Marine Science, Oban, Argyll PA37 1QA, UK; ^2^School of Life Science, Heriot-Watt University, Edinburgh EH14 4AS, UK

## Abstract

Coccolithophores are unicellular calcifying marine phytoplankton that can form large and conspicuous blooms in the oceans and make significant contributions to oceanic carbon cycling and atmospheric CO_2_ regulation. Despite their importance, the bacterial diversity associated with these algae has not been explored for ecological or biotechnological reasons. Bacterial membership of *Emiliania huxleyi* and *Coccolithus pelagicus* f. *braarudii* cultures was assessed using cultivation and cultivation-independent methods. The communities were species rich compared to other phytoplankton cultures. Community analysis identified specific taxa which cooccur in all cultures (*Marinobacter* and *Marivita*). Hydrocarbon-degrading bacteria were found in all cultures. The presence of Acidobacteria, Acidimicrobidae, *Schlegelella*, and *Thermomonas* was unprecedented but were potentially explained by calcification associated with coccolith production. One strain of Acidobacteria was cultivated and is closely related to a marine Acidobacteria isolated from a sponge. From this assessment of the bacterial diversity of coccolithophores, a number of biotechnological opportunities are evident, from bioprospecting for novel taxa such as Acidobacteria to helping understand the relationship between obligate hydrocarbonoclastic bacteria occurrence with phytoplankton and to revealing bacterial taxa that have a specific association with algae and may be suitable candidates as a means to improve the efficiency of mass algal cultivation.

## 1. Introduction

Marine phytoplankton are responsible for primary production in the oceans, using sunlight and inorganic nutrients to fix carbon dioxide that becomes the organic matter that supports the biological productivity of our oceans. Crucially, the availability of inorganic nutrients, such as N, P, and Si, necessary for phytoplankton growth, is indirectly controlled by a vast array of heterotrophic bacteria and Archaea that are responsible for remineralizing the organic matter produced by the phytoplankton. In this way, the fates of both phytoplankton and bacteria in the oceans are indirectly tied to one another. Additionally, some bacteria interact directly with phytoplankton cells in ways that can be beneficial or antagonistic. Overall, this makes the diversity of bacteria associated with phytoplankton of significant biotechnological interest for the discovery of novel and exploitable biodiversity and functions, such as tropodithietic acids produced by members of the* Roseobacter* clade [1], and, more recently, as a source of beneficial bacteria to promote and sustain mass algal culture systems [2, 3] that could be used to enhance the production of products such as pigments, lipids, and polysaccharides.

Remarkably, the bacterial diversity associated with one of the most important groups of marine phytoplankton, the coccolithophores, has not previously been investigated. This is surprising given the ecological importance of coccolithophorid algae, which by forming calcified scales (coccoliths) contribute significantly to the production of pelagic carbonate, as well as making a significant contribution to oceanic carbon cycling and regulation of atmospheric CO_2_ levels [4]. From a biotechnological perspective, these calcareous structures serve as solid surfaces on which bacterial colonisation can occur, which are very different mineralogically and biochemically from the organic or siliceous matrices of other phytoplankton such as dinoflagellates (e.g., cellulose armour) and diatoms (ridged silica structures). Together, these properties suggest that there may be associated bacterial taxa that are specific to coccolithophores, as the surface of coccoliths may facilitate the formation of complex bacterial communities containing unique biodiversity as well as a complex chemical signalling and secondary metabolite production [5].

Considerable effort is now being focused on the efficient cultivation of range of algae in closed growth systems, such as photobioreactors [6]. While mass culture of coccolithophorid algae is presently of modest biotechnological interest as a means of CO_2_ sequestration and lipid production [7], there is some indirect evidence that bacteria could be important to this process if it is to be developed because at least one species of coccolithophore,* Emiliania huxleyi*, has been shown to have an intimate reliance on bacterial presence [8]. Such observations are increasingly leading researchers to the suggestion that algal-associated bacteria should be considered for use in mass algal cultures systems [9, 10] and the broader realisation that there is a need to understand more about the ecological role of algal-associated bacteria, such as which taxa may be symbionts [2, 11] and the mechanisms by which they benefit their host [12, 13] and, ultimately, how to exploit this knowledge to improve the efficiency of mass algal culture systems.

The present study catalogued the cultivable and total bacterial diversity associated with laboratory cultures of* E. huxleyi* and* Coccolithus pelagicus* f.* braarudii* with the aim of uncovering associations and potential interactions between bacterial taxa and coccolithophores and generating a library of taxonomically defined cultivable bacteria for biotechnological exploitation. The study describes the total bacterial diversity of four coccolithophore cultures identified using a combination of 16S rRNA gene clone libraries and bacterial cultivation and fluorescence* in situ* hybridisation. The data revealed the presence of complex and taxonomically rich communities with a number of taxa that were unique to coccolithophores. The biotechnological potential of this diversity is discussed.

## 2. Materials and Methods

### 2.1. Algal Culture

Growth of all coccolithophore cultures (Table 1) was at 15°C in K medium diluted to (1/5)th full strength (K/5) [21] in 25 cm^2^ vented tissue culture flasks (Nunc) under cool-white fluorescent light of ca. 75 *μ*mol photons m^2^ s^1^ with a 12 : 12 light : dark (L : D) photoperiod. All cultures were actively calcifying. Cultures were handled aseptically to prevent bacterial contamination and cross-contamination between cultures.

The coccolithophore cultures used in this study (Table 1) were isolated using two different techniques. CCAP 920/8 was isolated by directly recovering a single calcifying cell from a water sample collected from a mesocosm experiment, using a glass capillary micropipette and growing them in an appropriate medium (J. Green pers. comm.). This mesocosm experiment (Bergen, Norway) used natural fjordic water collected and supplemented with nutrients [22, 23]. The RCC cultures were derived using a two-stage process initiated by addition of a small volume of water sample to culture media (e.g., K/5) to stimulate coccolithophore growth and, subsequently, isolate single calcifying cells by micropipette from patches of actively calcifying cells on the bottom of the growth vessel (RCC1200, RCC1203, RCC1214, and RCC1216; I. Probert pers. comm.) These cultures were derived from water samples collected as part of research cruises in oceanic and coastal regions of the Northern and Southern hemispheres. Further details can be obtained from the Roscoff Culture Collection (http://roscoff-culture-collection.org).

### 2.2. Bacterial Cultivation

Samples for molecular analysis were harvested by pipette from late* log* phase cultures that had been gently agitated to evenly suspend the nonmotile calcifying coccolithophores. A volume of the harvested cell suspension was serially diluted 10-fold and cell dilutions were plated onto ZM/10 agar (pH 7.8), a low organic concentration agar medium [24], and ONR7a [25] amended with trace metals and vitamins as used in ZM/10. ONR7a plates were amended with* n-*hexadecane soaked sterile filter paper. All plates were incubated in the dark at ca. 20°C for up to 8 weeks. Thereafter, unique colony morphologies were selected with the aid of a binocular microscope and were serially passaged on ZM/10 or ONR7a agar until a single colony morphotype was achieved. Several colonies of each morphology were then inoculated into 3 mL ZM/10 and ZM/1 [24] or for fastidious oil degraders and ZM/1 amended with 0.1% sodium pyruvate and grown with gentle shaking (ca. 20°C) until turbidity was visible. From each broth culture 1-2 mL was harvested for DNA extraction, and 1 mL was amended with sterile glycerol (20% v/v) and frozen at −80°C.

### 2.3. 16S rRNA Gene Analysis

16S rRNA gene clone libraries were constructed from 1 mL of the suspended late* log* phase culture (as above). Bacterial and algal cells were harvested by centrifugation (13,000 ×g for 10 min), the spent medium discarded and the cell pellets stored frozen at −80°C until DNA was extracted. DNA extraction used a cetyltrimethylammonium bromide purification method [26] amended to suspend the cell pellet in 100 mM Tris-HCl (pH 8.0), 150 mM NaCl, and 10 mM EDTA, to which lysozyme (5 mg mL^−1^ final concentration) was then added and incubated at 37°C for 30 min. Bacterial 16S rRNA gene sequences were amplified by the PCR from extracted DNA based on the universal bacterial primers 27f and 1492r [27], except that 27f primer was modified to include the underlined 5′ adapter sequence (27f adapter; CTAATACGACTCAGCTATGCACTAGRGTTTGATCMTGGCTCAG). The PCR reaction contained a final concentration of 1.8 mM Mg^2+^, 0.5 *µ*M of each primer, 1 U Taq polymerase and 1x PCR buffer (New England Biolabs). The amplification protocol was 94°C for 5 min, followed by 20 cycles of 55°C for 30 s, 72°C for 3 min, and 94°C for 10 s, followed by 72°C for 10 min. For each coccolithophore culture, the amplicons from three independent 50 *μ*L PCR reactions were pooled and purified with Montage PCR filters (Millipore) and then cloned using the pGEM-T Easy vector kit (Promega). 16S clones from each library were picked by sterile toothpick and reamplified using the forward adapter primer sequence (27fSeqAdapter; CTAATACGACTCAGCTATGCACT) and 1492r. This primer combination prevents amplification of the* Escherichia coli* DH5*α* host 16S rRNA gene. Amplicon products were cleaned using shrimp alkaline phosphatase (Promega) and exonuclease I (New England Biolabs) and then sequenced using BigDye version 3.1 terminator chemistry (Applied Biosystems) primed using the 27fSeqAdapter oligonucleotide. DNA sequence products were called on an ABI3730 instrument (Applied Biosystems). The method of DNA extraction, the primer combination of 27f and 1492r, and DNA sequencing were used for cultivable bacterial strains (cloning was omitted and the products of a single PCR amplification were submitted for DNA sequencing as above).

### 2.4. Phylogenetic Analysis

Bacterial 16S rRNA gene sequences were classified using the RDP II Classifier [15]. Where clone library operational taxonomic units (OTUs) corresponded to cultivable strains (>99% identity) from the same coccolithophore culture, the cultivable strain number was used to denote the OTU. All 16S sequences were screened for chimeric sequences using Bellerophon [28]. Phylogenetic inference was performed using the ARB software suite [29]. Alignments were built using NAST aligner [30] and imported into ARB and corrected as necessary. Tree constructions used a masked alignment (lanePH) and the maximum likelihood model as implemented in PhyML [31]. UniFrac analysis [32] was used to generate various statistics and principal coordinate analysis (PCoA) based on the inferred ARB PhyML tree and an unweighted dataset (i.e., not weighted for species abundance). Analysis of the clone libraries at varying levels of OTU clustering, rarefaction, and reclassification of OTU identities were performed using MOTHUR [14]. 16S rRNA gene sequences are available with the following accession numbers: EF140750-EF140751, EF140753-EF140754, EU052756, EU052761-EU052762, EU052764-EU052765, EU732746, KC295293-KC295413, and KM279011-KM279029. All cultivable strain and clone 16S sequence data were generated and sequenced in 2008, but 16S data were submitted at later dates in relationship to specific manuscript submissions.

### 2.5. Fluorescence* In Situ* Hybridisation (FISH)

Cy3 labelled Acidobacteria group specific probe SS_HOL1400 [33] and EUB338 [34] were used in this study. The SS_HOL1400 probe was confirmed by pairwise alignment to have a 100% match to the two Acidobacteria identified in this study (DG1540 and OTU AC472_G8). Briefly, coccolithophore cultures were gently suspended prior to removing ca. 1 mL that was then fixed with formaldehyde (3% final concentration) for 1 hr in the dark at room temperature. Volumes of 10 to 100 *μ*L of fixed cells were then filtered onto 25 mm 0.2 *μ*m track-etched white polycarbonate filters and washed with 10 mL of 0.2 *μ*m filtered deionised water. Filters were air dried, dehydrated in ethanol (50, 80, 100%), and air dried and stored at −20°C. Hybridisation and washing proceeded as described [33, 35]. Hybridised filters were mounted with Vectashield which contains the DNA stain DAPI and antifade agent and viewed by epifluorescence microscopy in the normal way using Axioskop 2plus and images captured using AxioCam HRc and processed using AxioVision (Zeiss) and Cy3 and DAPI composite epifluorescent images compiled and montaged using Adobe Photoshop CS4.

## 3. Results and Discussion

### 3.1. Community Analysis

Five cultures were sampled for cultivable bacteria and cell pellets frozen at −80°C prior to 16S rRNA gene clone library construction and analysis. Clone libraries were constructed from 16S amplicons produced from a total of 20 thermal cycles to reduce the potential for PCR artefacts and heteroduplexes [36]. A total of 316 clones from four of the five libraries were sequenced leading to the identification of 85 bacterial operational taxonomic units (OTUs) representing five bacterial phyla (Table 2). The Chao-1 estimator of species richness indicated two of the libraries to have ca. 100 phylotypes. Bacterial cultivation using a low organic strength marine agar and extended incubation periods of up to eight weeks were used and increased the overall assessment of total species richness by ca. 33% compared to clone library data alone. Cultivation identified a total of 105 unique isolates (Table 3), and, in comparison to clone library data, cultivation success varied across the different phyla, with ca. 77% of Alphaproteobacteria, 62% of Gammaproteobacteria, and 55% of Bacteroidetes being cultivable. Overall, based on the cultures with clone and cultivation data, a total of 127 phylotypes were identified which results in an average species richness of 32 phylotypes per culture (Table 2).

The bacterial species richness of the coccolithophore cultures was higher than the average range of 17–19 phylotypes per dinoflagellate culture [37, 38] where the same methods were used, and higher than the 9–14 phylotypes that were identified in diatom cultures [39–41], although diatom analyses used denaturing gradient gel electrophoresis which may underestimate species richness compared to clone libraries. Nevertheless, in comparison to dinoflagellates, coccolithophores appear to maintain a more species rich bacterial community. This increased richness could have originated from the presence of higher levels of bacteria attached to coccolithophorid cells when they were isolated from field material. This may be an intrinsic property of the cell surface (e.g., coccolith production) or it could be linked to the method of culture isolation from the field (see materials and methods). Alternatively, it may be because actively growing and calcifying coccolithophores create a more complex biochemical and biophysical environment than do dinoflagellates (e.g., algal exudates, acidic polysaccharides, calcite, and increased surface area), which supports the greater diversity of bacteria observed. Illustrating the role calcified cell plates can have as an attachment point is a report describing the attachment of a nitrogen-fixing cyanobacterial symbiont to its calcifying picoeukaryote host [42].

**Figure 1:**
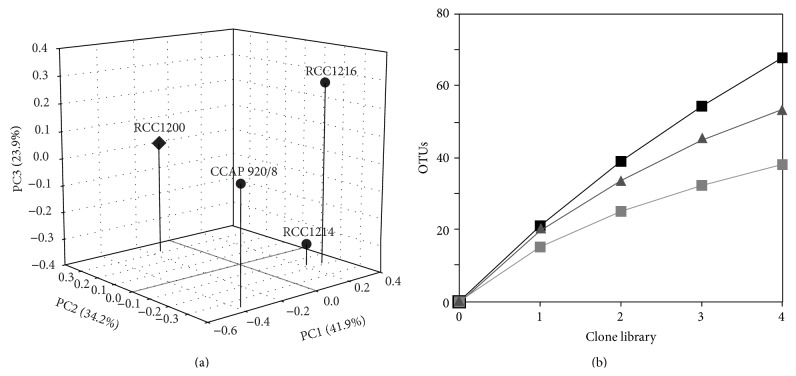
Total bacterial community analysis. (a) Unweighted UniFrac principal coordinate analysis of the total bacterial diversity of* E. huxleyi* (●) and* C. pelagicus* f.* braarudii* (*◆*) cultures. (b) Rarefaction analysis of shared 16S clone library diversity at OTU clustering levels of 0.01 (black ■), 0.05 (grey ▲), and 0.1 (grey ■) was based on an average of 79 sequenced clones per library.

UniFrac analysis did not detect any significant differences in pairwise comparisons between the total unweighted diversity of the four communities (OTU and cultivable; *P* = 1.0), although each community was clearly separated by UniFrac PCoA (Figure 1(b)). Rarefaction analysis of the shared diversity showed that, at the 0.01 OTU identity level, the rate of new diversity discovered was still increasing (Figure 1(a)), but that at the level of ca. family and below (≤0.10) the amount of shared diversity was nearing an asymptote (Figure 1(a)). This indicates that the communities contained a high proportion of unique species-level microdiversity [36], while the community taxonomic composition at and below the family level is conserved across the cultures. The broad diversity observed may be a product of taxonomically similar groups of bacteria being adapted to or are selected by the coccolithophores. Whereas the fine scale microdiversity seen in the 16S rRNA sequences could possibly be due to the different geographic origins of the cultures (Table 1) or selection of different ecotypes based on adaption to the coccolithophore strains or neutral sequence variation. The origin of this 16S rRNA variation is unknown but could be revealed by more in-depth analyses of a greater number and range of coccolithophore species as well as analysis of genomic variation in some of the diverse clusters [36].

**Figure 2:**
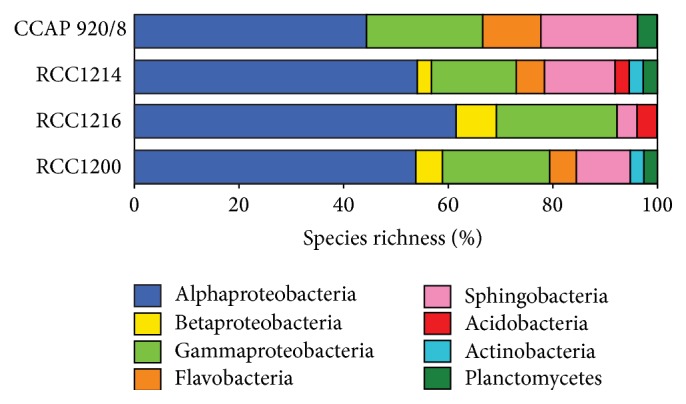
Taxonomic composition of the total bacterial community. Percentage species richness was based on the number of unique clone library and cultivable phylotypes identified in each culture.

The bacterial diversity of coccolithophore cultures was spread across five phyla (Figure 2). Species richness was dominated by Alphaproteobacteria (average 53%), of which just under half (43%) belonged to the* Roseobacter* clade. Gammaproteobacteria (21% on average) and Bacteroidetes (17% on average) comprised the next most prominent taxonomic groups present in all the cultures. Within the Bacteroidetes, Sphingobacteria were present in all cultures, but Flavobacteria were present in only three cultures and were relatively species poor. Betaproteobacteria and Planctomycetes were each identified in three of four cultures. Actinobacteria and Acidobacteria were each present in two cultures. The broad taxonomic composition was similar to that of dinoflagellates [37, 43–45] and diatoms [40, 41, 46] where Alphaproteobacteria dominate, with varying numbers of Bacteroidetes and Gammaproteobacteria. Qualitatively, the gammaproteobacterial composition in the coccolithophore cultures bore a greater similarity to that observed with dinoflagellates than that from diatoms. For example,* Marinobacter*,* Alcanivorax,* and oligotrophic marine Gammaproteobacteria- (OMG-) like bacteria were more common in coccolithophore as well as dinoflagellate cultures [12, 37], whereas diatom gammaproteobacterial diversity is more typically dominated by* Pseudoalteromonas* and* Alteromonas* [39, 40]. A notable difference of the coccolithophore cultures to that of dinoflagellates and diatoms was that Bacteroidetes diversity of the coccolithophores was dominated by Sphingobacteria (Figure 2) and not Flavobacteria, the latter being typically more prevalent with dinoflagellates and diatoms [47]. This shift in laboratory culture diversity may be a reflection of natural coccolithophore blooms, as the most abundant bacterial taxa (~19%) in the coccolithophore-attached fraction recovered from the Bay of Biscay were Sphingobacteria [48].

### 3.2. Cooccurring Bacterial Taxa: Marivita and Marinobacter

**Figure 3:**
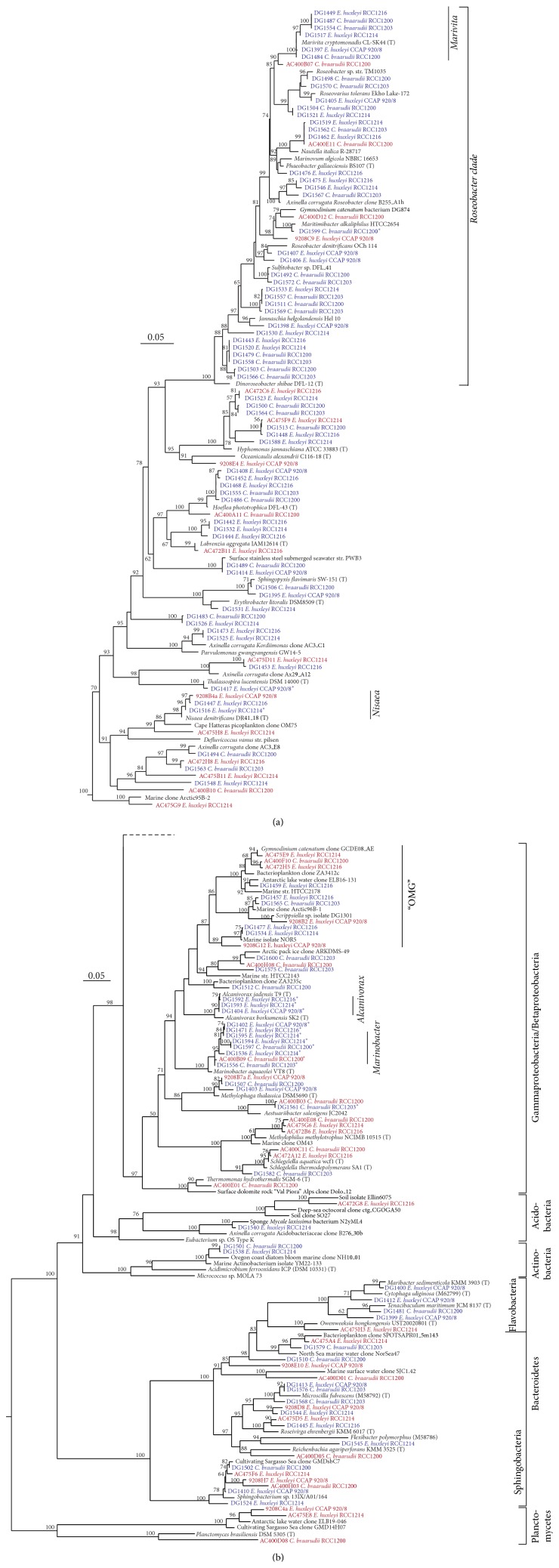
Phylogenetic affiliation (16S rRNA gene) of coccolithophore-associated bacteria, depicting (a) Alphaproteobacteria and (b) Gammaproteobacteria, Betaproteobacteria, Acidobacteria, Actinobacteria, Bacteroidetes, and Planctomycetes. Dendrograms were constructed using a lanePH filtered alignment using maximum likelihood and the HKY model of nucleotide substitution (PhyML). Bootstrap support ≥50% support for the branching is shown. ∗ denotes strains shown to use* n*-hexadecane as a sole carbon source. Clones (red font) and strains (blue font) identified in this study and representative clones or strains from public databases (black font). Scale bar: 0.05 nucleotide substitutions per base.

Phylotypes belonging to two phylogenetic clusters were observed in all five cultures. Strains in the first cluster (DG1397, 1449, 1484, 1487, 1517, and 1554) were closely related (≥98.2% identity) to the type strains of the genus* Marivita* (Figure 3(a)). To date, there is relatively little known about the genus Marivita, but related phylotypes have been identified with a range of algal cultures [24, 38, 49, 50], as well as representing ca. 4–6% of the phylotype abundance in summer and autumn clone libraries from productive surface waters off the north west of Spain [51]. In other work, we have observed that strains of* Marivita* (as well as other taxa) act as dinoflagellate symbionts promoting dinoflagellate growth (D. Green and C. J. S. Bolch, publication in prep.). Collectively, this suggests that* Marivita* may have a specific adaptation to associating with phytoplankton, including coccolithophores, and could be analogous to the other Roseobacters that are regarded as algal symbionts, such as* Dinoroseobacter shibae* [52].

The second phylogenetic cluster was cultivable isolates belonging to the gammaproteobacterial genus* Marinobacter* (Figure 3(b)).* Marinobacter* have been found with a range of algae, principally dinoflagellates [37, 38, 43, 45, 53] and some diatoms [12]. Despite an apparent algal culture association, their abundance in the marine environment is typically low; for example, the multiyear average in the Western English Channel was ca. 0.18% (min. 0.00% and max. 4.5%) of total pyrosequencing reads [19]. One explanation for this disparity could be that the frequent association with laboratory cultures represents a laboratory-induced artefact caused by algal cultivation that is selecting for* Marinobacter*, possibly based on their utilisation of algal aliphatics. However, their markedly lower frequency in diatom cultures (~22%, as compared to ≥83% in coccolithophore and dinoflagellate cultures; [12]) argues against their presence being a simple laboratory artefact because algal culture media and methodology are similar for all three algal lineages and this should not cause such a bias. We speculate that* Marinobacter* have a specific adaptation to coccolithophores and dinoflagellates. As* Marinobacter* have been recognised in the context of algal-bacterial interactions, such as increasing iron bioavailability to dinoflagellates [12], promoting growth of the dinoflagellate* G. catenatum* [54] and the cyanobacterium* Prochlorococcus* [55], this provides some support to the hypothesis that this association represents a specific adaptation to life with these algae.

In the context of applying bacteria to improve the mass cultivation of algae, the evidence from this study and others point toward* Marivita* and* Marinobacter* as two candidate bacterial groups that have some potential because of their common cooccurrence with algal cultures, as well as evidence that strains of both genera can have beneficial effects such as growth promotion. Furthermore, neither genus has been linked to antagonistic interactions with algae that we are aware of.

### 3.3. Hydrocarbon-Degrading Bacteria

In the present study, we sought to identify whether there were hydrocarbon-degrading bacteria present in these cultures, as we have previously observed an association with algae [24]. We identified and confirmed that all the* Alcanivorax* and* Marinobacter* strains that were isolated could utilise* n*-hexadecane as the sole carbon and energy source (Figure 3). This is consistent with a number of reports showing that both genera are important and are often highly abundant in the marine environment during oil spill events (e.g. [56]). Four strains that did not belong to these two genera were also shown to use* n-*hexadecane: one was a member of the Alteromonadaceae (DG1561), three were members of Alphaproteobacteria belonging to* Maritimibacter* of the* Roseobacter* clade (DG1599), and* Thalassospira* (DG1417) and* Nisaea* (DG1516) both belonged to the Rhodospirillaceae. This study and others reporting the coassociation of oil-degrading bacteria and algae [24, 57–59] suggest that there may be a specific association between the two. The simplest explanation for this may reflect the fact that many microalgae are lipid-rich [60] and that, in the natural environment, algal cells provide an abundant source of energy-rich aliphatic compounds available to hydrocarbonoclastic bacteria.

The presence of hydrocarbon-degrading bacteria living with algae presents a number of biotechnological opportunities. First, we believe that it is important to understand their ecological relationship to algae in the marine environment and then to use this knowledge to try improving natural oil spill bioremediation: for example, will there be better natural bioremediation of oil spills in regions that are dominated by primary production? Or is the use of iron fertilization during oil spill events beneficial in stimulating both primary production and the associated bacterial community that clearly comprises many key hydrocarbonoclastic bacteria such as* Alcanivorax*, as well as ensuring a sufficient supply of iron for the iron-requiring alkane hydroxylases of all oil-degrading bacteria [61]? Second, specific genes and activities of oil-degraders can potentially be exploited for biosurfactant, bioemulsifier [62, 63], or polyhydroxyalkanoate production [64], or the potential development of alkane hydroxylases to catalyze difficult-to-synthesize molecules [65]. While hydrocarbon-degrading strains from coccolithophores have not been explored for polycyclic aromatic hydrocarbon (PAH) degradation, related research with other algae shows that they do harbor highly fastidious PAH degrading bacteria [58, 59]. Overall, coccolithophore and other phytoplankton cultures are a useful starting point for bioprospecting for specialist hydrocarbon-degrading microorganisms. They may also be used as model systems to study the ecological underpinnings of this interrelationship, with the aim to use this knowledge to help enhance oil bioremediation.

### 3.4. Atypical Bacterial Taxa

A number of the phylotypes identified were not typical of bacterial taxa found previously with algal cultures. First was the identification of two phylotypes belonging to the phylum Acidobacteria (Figure 3(b)), which have not previously been identified in any algal culture. OTU AC472G8 was affiliated to Group IV Acidobacteria, which appear to be rare in the marine environment (e.g., the closest marine phylotypes is DQ071127; 95.6% id). The other Acidobacteria, DG1540 (KC295408), was affiliated to the Holophagales (group VIII). This isolate was cultivated on the low organic strength marine agar ZM/10 at ca. pH 7.8. Subsequent cultivation experiments with this strain demonstrated that it did not grow at pH values below pH 7.0 but grew abundantly up to pH 9.5, the highest pH tested (data not shown). This indicates that while it is related to the phylum Acidobacteria, it is not acidophilic as is characteristic of this phylum. The closest 16S rRNA gene sequences to DG1540 were all marine and belonged to coral-associated bacterial clones (FJ202764.1, FJ203188.1; 89.3 and 88.4% id resp.), a sponge-associated bacterial isolate (EF629834; 95.6% id), and* Acanthopleuribacter pedis* (AB303221; 88.6% id) isolated from a marine chiton [66]. The second atypical taxon was two cultivable Actinobacteria strains (DG1501 and DG1538) affiliated to the family Acidimicrobidae. A single Acidimicrobidae OTU (DQ376149; 97.0% id) has previously been identified in a diatom culture. Related Acidimicrobidae OTUs and isolates have been found in a range of marine samples (Table 4), including corals (GU118194; ca. 92.9% id), sponges (EU236418; ca. 94.8% id), marine sediment (AB286031; ca. 96.8% id), and surface waters (GQ850547 and DQ372838; ca. 98.1 and 94.5% id resp.). Third, a gammaproteobacterial OTU affiliated to* Thermomonas* was identified in one culture only. This genus has been identified in thermal springs [67] and at several oceanic stations and in microbial mat communities (Table 4). Fourth, two OTUs belonging to the genus* Schlegelella* (Betaproteobacteria) were identified in two separate cultures. Like* Thermomonas*, this genus has been observed in mineral springs [67, 68] and has been recorded in the marine environment from the same sites as* Thermomonas* (Table 4). Finally, three very closely related OTUs (≥97.5% ID) affiliated to the Methylophilaceae (Betaproteobacteria) were identified but not cultured. These OTUs are distantly related to the well-described OM43 clade (≥87.9% ID) of obligate methylotrophic bacteria linked with phytoplankton blooms [69], and, to our knowledge, only a single related OTU has previously been identified in an algal culture (KEppi37, AF188168; [43]).

**Figure 4:**
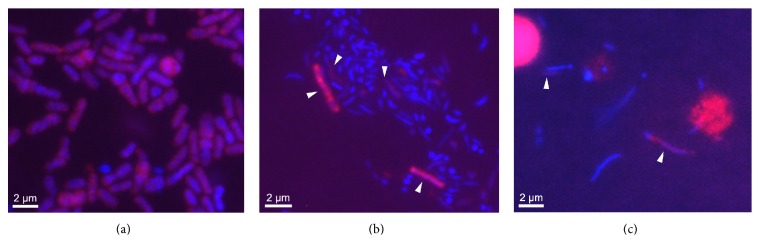
Composite DAPI-Cy3 FISH detection of Acidobacteria in coccolithophore cultures. (a) Positive control DG1540 (isolated from* E. huxleyi* RCC1214). (b)* E. huxleyi* RCC1214 culture and (c)* E. huxleyi* Bergen_05_08 (Bergen mesocosm, 2008). Arrowheads indicate Acidobacteria cells present on the DAPI-Cy3 composite images. Scale bar: 2 *μ*m.

As the observation of Acidobacteria in algal cultures was apparently unusual, an additional three* E. huxleyi* cultures were screened using a phylum-level Acidobacteria FISH probe, these were LY1_05 (LY1 2005, Oban, Scotland), Bergen_05_08 (Bergen Mesocosm, 2008, Norway), and CS_08 (Celtic Sea 2008, UK). The positive control, DG1540 (Figure 4(a)), was identified in RCC1214 from where it had originally been isolated (Figure 4(b)). However, Acidobacteria were not detected in RCC1216 from which the group IV acidobacterial OTU AC472G8 had been identified. Acidobacteria were detected in the Bergen mesocosm culture isolated in 2008 (Bergen _05_08) (Figure 4(c)).

The presence of Acidobacteria (DG1540 and AC472G8, as well as FISH detection in one other* E. huxleyi* culture; Figure 4) and the Acidimicrobidae (Actinobacteria) led us to consider why putatively acidophilic bacteria were associated with* E. huxleyi*. We speculate that this may reflect a pattern of finding marine Acidobacteria associated with calcareous organisms or structures such as the chiton [66], corals [70], and stromatolites [71]. The reasons for their association with carbonate structures are unclear, but these organisms may be involved in catabolizing the organic matrix associated with the coccoliths and potentially driving calcite dissolution through acid production [72]. The observation of* Schlegelella* and* Thermomonas* in the cultures may also be attributable to calcification in the cultures because both* Schlegelella* and* Thermomonas* OTUs have been isolated from thermal spring waters containing a high mineral and carbonate loading [67, 68]. As both genera have been identified by pyrosequencing from marine environmental samples (Table 4), this suggests that their presence could be related to the high mineral content of calcifying coccolithophores and not the product of external contamination of the cultures.

The lack of phylogenetic coherence amongst the atypical taxa detected does not appear to be an ecological pattern, but we suggest that, from the biochemical associations of related taxa to those detected in this study (Figure 3(b)), this points at carbonate chemistry as the most likely reason explaining their presence. This may also explain why these taxa have not previously been identified with diatoms and dinoflagellates, because the latter are not calcifying organisms. Thus, we speculate that the atypical taxa such as Acidobacteria,* Schlegelella,* and* Thermomonas* are evidence that actively calcifying coccolithophores chemically and physically structure the bacterial community to include organisms adapted to calcareous material. This is analogous to the way in which diatom exudates structure the bacterial associates in culture [40] and of estuarine sediments [73].

Cultivation of DG1540 and related Acidobacteria (N2yML4; EF629834) from sponge aquaculture [74] represent a potentially valuable biotechnological and ecological resource because of their taxonomic relationship to prevalent groups of bacteria in sponge and coral microbial communities [20, 75]. Whether or not these Acidobacteria produce secondary metabolites is of clear interest given the importance of the marine sponge microbiome for production of a wide range of novel chemical entities [76]. However, cultivation of these and other marine Acidobacteria has clearly been sporadic (totalling three: this study, Mohamed et al. 2008 [74], and Fukunaga et al. 2008 [66]) despite the many efforts to cultivate sponge and coral bacteria. This indicates a need to develop different primary isolation media and strategies to improve discovery of these and related Acidobacteria, as well as the other atypical taxa identified in this study. The inclusion of solid carbonate (or siliceous) material in agar or in liquid enrichment media may be one starting point; and addition of 5% CO_2_ (v/v) has been successfully used to improve isolation of soil Acidobacteria [77]. The question of whether all or any marine Acidobacteria are acidophilic is unclear given that ambient seawater pH is typically nearer to pH 8 and the evidence that DG1540 would not grow below pH 7. Genomic analysis of DG1540 is now underway, and this data will be mined to address questions of their ecological, metabolic and biotechnological potential, as well as contribute to cultivation efforts of related marine Acidobacteria found with corals and sponges.

## 4. Conclusions

The present study is the first to catalogue the bacterial diversity associated with two important species of coccolithophorid phytoplankton. It revealed that both* E. huxleyi* and* C. pelagicus* f.* braarudii* cultures had relatively species rich bacterial communities compared to dinoflagellates. It identified a number of bacterial taxa not previously detected in other phytoplankton cultures and included the unexpected finding of a number of putatively acidophilic bacteria. Overall, this is suggested to signify that coccolithophores possess a greater range of available niches, as well as novel niches, compared to other kinds of phytoplankton such as dinoflagellates and diatoms. It is proposed that the presence of some of the atypical taxa may represent selection of specific bacterial taxa adapted to the active calcification occurring in these coccolithophore cultures. The cultures were observed to share bacterial diversity with other algal cultures, most notably, dinoflagellates. Two genera,* Marinobacter* and* Marivita*, were observed to occur in all of the coccolithophore cultures, and whilst these genera may be a product of laboratory culture induced artefacts, closely related bacteria have been shown to benefit dinoflagellate and* Prochlorococcus* growth, suggesting that the common occurrence of these bacteria may represent a specific interdependence between the bacteria and algae. Hydrocarbon-degrading bacteria were found in all cultures, and, most notably, each* E. huxleyi* culture had a closely related strain of* Alcanivorax*, a well-known and highly fastidious oil-degrading bacterium.

Knowledge of the biodiversity contained within these coccolithophore cultures has a number of potential uses, that include the types of bacteria that could be employed to improve the efficiency of mass cultivation of marine algae, and, as a model system to help uncover the ecological relationship of why oil-degrading bacteria cooccur with phytoplankton, and how this knowledge may be used to improve oil-spill bioremediation. Furthermore, the cultivation of a marine bacterium related to Acidobacteria found with marine sponges represents a rare opportunity to understand more about the ecology of these bacteria and to explore the biotechnological potential this may entail.

## Figures and Tables

**Table 1:** Coccolithophorid cultures examined in this study.

Coccolithophore^1^	Origin	Location	Isolation date	Culture collection
*E. huxleyi *				
CCAP 920/8	Bergen mesocosm, Norway	Coastal	1992	CCAP
RCC 1214	Bay of Napoli, Italy	Coastal	2000	Roscoff
RCC 1216	Tasman Sea, New Zealand	Oceanic	1998	Roscoff
*C. pelagicus* f.* braarudii *				
RCC 1200	South Atlantic, Namibia	Oceanic	2000	Roscoff
RCC 1203	Bay of Biscay, North Atlantic	Coastal	1999	Roscoff

^1^Cultures are also known as CCAP 920/8 = PLY B92/11; RCC 1214 = AC475; RCC 1216 = AC472; RCC 1200 = AC400; RCC 1203 = AC392.

**Table 2:** Bacterial species richness deduced from cultivation and clone library studies.

Coccolithophore	OTUs^1^				Cultivable	Total
	No.	Chao-1	Shannon	Evenness		
*E. huxleyi *						
CCAP 920/8	17	18	2.61	0.9212	17	27
RCC 1214	25	103	2.84	0.8823	25	35
RCC 1216	15	18	2.28	0.8419	19	26
*C. pelagicus* f.* braarudii *						
RCC 1200	28	96	2.76	0.8282	23	39
RCC 1203	ND	ND	ND	ND	21	—

^1^Number of OTUs, Chao-1, and Shannon index calculated at 0.01 distance as calculated in MOTHUR [14]. Evenness was calculated as (*J*′) = *H*′/ln⁡(S), where *H*′ is Shannon diversity index; *S* is species richness. ND: not determined.

**Table 3:** Bacterial isolates cultivated from *E.  huxleyi* and *C.  pelagicus *f.*  braarudii*.

Strain number	Host	Accession	Taxonomy^1^			
			Phylum/class	Family	Genus	Conf.
	*E. huxleyi*					

DG1395	CCAP 920/8	KC295338	Alphaproteobacteria	Sphingomonadaceae	*Sphingorhabdus *	99
DG1397	CCAP 920/8	KC295339	Alphaproteobacteria	Rhodobacteraceae	*Marivita *	100
DG1398	CCAP 920/8	KC295340	Alphaproteobacteria	Rhodobacteraceae	*Loktanella *	52
DG1399	CCAP 920/8	KC295341	Flavobacteria	Flavobacteriaceae	*Aureitalea *	60
DG1400	CCAP 920/8	KC295342	Flavobacteria	Flavobacteriaceae	*Maribacter *	100
DG1402	CCAP 920/8	EF140754	Gammaproteobacteria	Alteromonadaceae	*Marinobacter *	100
DG1403	CCAP 920/8	KC295343	Gammaproteobacteria	Piscirickettsiaceae	*Methylophaga *	100
DG1404	CCAP 920/8	KC295344	Gammaproteobacteria	Alcanivoracaceae	*Alcanivorax *	100
DG1405	CCAP 920/8	KC295345	Alphaproteobacteria	Rhodobacteraceae	*Roseovarius *	100
DG1406	CCAP 920/8	KC295346	Alphaproteobacteria	Rhodobacteraceae	*Sulfitobacter *	98
DG1407	CCAP 920/8	KC295347	Alphaproteobacteria	Rhodobacteraceae	*Sulfitobacter *	32
DG1408	CCAP 920/8	KC295348	Alphaproteobacteria	Phyllobacteriaceae	*Hoeflea *	100
DG1410	CCAP 920/8	KC295349	Sphingobacteria	Chitinophagaceae	*Balneola *	100
DG1412	CCAP 920/8	KC295350	Flavobacteria	Flavobacteriaceae	*Arenibacter *	100
DG1413	CCAP 920/8	KC295351	Sphingobacteria	Flammeovirgaceae	*Marinoscillum *	100
DG1414	CCAP 920/8	KC295352	Alphaproteobacteria	Rhodobacteraceae	*Ahrensia *	35
DG1417	CCAP 920/8	EU052756	Alphaproteobacteria	Rhodospirillaceae	*Thalassospira *	100

DG1442	RCC 1216	KC295353	Alphaproteobacteria	Phyllobacteriaceae	*Ahrensia *	29
DG1443	RCC 1216	KC295354	Alphaproteobacteria	Rhodobacteraceae	*Roseovarius *	47
DG1444	RCC 1216	KC295355	Alphaproteobacteria	Rhodobacteraceae	*Ahrensia *	42
DG1445	RCC 1216	KC295356	Sphingobacteria	Flammeovirgaceae	*Fabibacter *	82
DG1447	RCC 1216	KC295357	Alphaproteobacteria	Rhodospirillaceae	*Nisaea *	100
DG1448	RCC 1216	KC295358	Alphaproteobacteria	Hyphomonadaceae	*Hyphomonas *	73
DG1449	RCC 1216	KC295359	Alphaproteobacteria	Rhodobacteraceae	*Marivita *	100
DG1452	RCC 1216	KC295360	Alphaproteobacteria	Phyllobacteriaceae	*Hoeflea *	100
DG1453	RCC 1216	KC295361	Alphaproteobacteria	Rhodobiaceae	*Roseospirillum *	37
DG1457	RCC 1216	KC295362	Gammaproteobacteria	Oceanospirillaceae	*Oceaniserpentilla *	20
DG1459	RCC 1216	KC295363	Gammaproteobacteria	Gammaproteobacteria	Unclassified	—
DG1462	RCC 1216	KC295364	Alphaproteobacteria	Rhodobacteraceae	*Roseovarius *	52
DG1468	RCC 1216	KC295365	Alphaproteobacteria	Phyllobacteriaceae	*Hoeflea *	100
DG1471	RCC 1216	KC295366	Gammaproteobacteria	Alteromonadaceae	*Marinobacter *	100
DG1473	RCC 1216	KC295367	Alphaproteobacteria	Kordiimonadaceae	*Kordiimonas *	100
DG1475	RCC 1216	KC295368	Alphaproteobacteria	Rhodobacteraceae	*Roseovarius *	91
DG1476	RCC 1216	KC295369	Alphaproteobacteria	Rhodobacteraceae	*Phaeobacter *	100
DG1477	RCC 1216	KC295370	Gammaproteobacteria	Gammaproteobacteria	*Congregibacter *	100
DG1592	RCC 1216	EU052761	Gammaproteobacteria	Alcanivoracaceae	*Alcanivorax *	100

DG1516	RCC 1214	KC295392	Alphaproteobacteria	Rhodospirillaceae	*Nisaea *	100
DG1517	RCC 1214	KC295393	Alphaproteobacteria	Rhodobacteraceae	*Marivita *	100
DG1519	RCC 1214	KC295394	Alphaproteobacteria	Rhodobacteraceae	*Oceanicola *	65
DG1520	RCC 1214	KC295395	Alphaproteobacteria	Rhodobacteraceae	*Roseovarius *	47
DG1521	RCC 1214	KC295396	Alphaproteobacteria	Rhodobacteraceae	*Roseovarius *	100
DG1523	RCC 1214	KC295397	Alphaproteobacteria	Hyphomonadaceae	*Hyphomonas *	100
DG1524	RCC 1214	KC295398	Sphingobacteria	Chitinophagaceae	*Balneola *	83
DG1525	RCC 1214	KC295399	Alphaproteobacteria	Kordiimonadaceae	*Kordiimonas *	100
DG1526	RCC 1214	KC295400	Alphaproteobacteria	Sneathiellaceae	*Oceanibacterium *	44
DG1530	RCC 1214	KC295401	Alphaproteobacteria	Rhodobacteraceae	*Oceanicola *	14
DG1531	RCC 1214	KC295402	Alphaproteobacteria	Erythrobacteraceae	*Altererythrobacter *	96
DG1532	RCC 1214	KC295403	Alphaproteobacteria	Rhodobacteraceae	*Ahrensia *	35
DG1533	RCC 1214	KC295404	Alphaproteobacteria	Rhodobacteraceae	*Loktanella *	67
DG1534	RCC 1214	KC295405	Gammaproteobacteria	Gammaproteobacteria	*Congregibacter *	100
DG1536	RCC 1214	KC295406	Gammaproteobacteria	Alteromonadaceae	*Marinobacter *	100
DG1538	RCC 1214	KC295407	Actinobacteria	Acidimicrobiaceae	*Ilumatobacter *	100
DG1540	RCC 1214	KC295408	Acidobacteria	Acanthopleuribacteraceae	*Acanthopleuribacter *	100
DG1544	RCC 1214	KC295409	Sphingobacteria	Flammeovirgaceae	*Echidna *	100
DG1545	RCC 1214	KC295410	Sphingobacteria	Cytophagaceae	*Leadbetterella *	82
DG1546	RCC 1214	KC295411	Alphaproteobacteria	Rhodobacteraceae	*Roseovarius *	82
DG1548	RCC 1214	KC295412	Alphaproteobacteria	Rhodospirillaceae	*Tistlia *	31
DG1588	RCC 1214	KC295413	Alphaproteobacteria	Hyphomonadaceae	*Hyphomonas *	93
DG1593	RCC 1214	EU052762	Gammaproteobacteria	Alcanivoracaceae	*Alcanivorax *	100
DG1594	RCC 1214	EF140750	Gammaproteobacteria	Alteromonadaceae	*Marinobacter *	100
DG1595	RCC 1214	EF140751	Gammaproteobacteria	Alteromonadaceae	*Marinobacter *	100

	*C. pelagicus *f.* braarudii *					

DG1479	RCC 1200	KC295371	Alphaproteobacteria	Rhodobacteraceae	*Oceanicola *	70
DG1481	RCC 1200	KC295372	Flavobacteria	Flavobacteriaceae	*Tenacibaculum *	81
DG1483	RCC 1200	KC295373	Alphaproteobacteria	Sneathiellaceae	*Oceanibacterium *	51
DG1484	RCC 1200	KC295374	Alphaproteobacteria	Rhodobacteraceae	*Marivita *	100
DG1486	RCC 1200	KC295375	Alphaproteobacteria	Phyllobacteriaceae	*Hoeflea *	100
DG1487	RCC 1200	KC295376	Alphaproteobacteria	Rhodobacteraceae	*Marivita *	100
DG1489	RCC 1200	KC295377	Alphaproteobacteria	Rhodobacteraceae	*Ahrensia *	46
DG1492	RCC 1200	KC295378	Alphaproteobacteria	Rhodobacteraceae	*Sulfitobacter *	64
DG1494	RCC 1200	KC295379	Alphaproteobacteria	Rhodospirillaceae	*Magnetospira *	73
DG1498	RCC 1200	KC295380	Alphaproteobacteria	Rhodobacteraceae	*Roseovarius *	100
DG1500	RCC 1200	KC295381	Alphaproteobacteria	Hyphomonadaceae	*Hyphomonas *	100
DG1501	RCC 1200	KC295382	Actinobacteria	Acidimicrobidae	*Ilumatobacter *	100
DG1502	RCC 1200	KC295383	Sphingobacteria	Chitinophagaceae	*Balneola *	100
DG1503	RCC 1200	KC295384	Alphaproteobacteria	Rhodobacteraceae	*Jannaschia *	40
DG1504	RCC 1200	KC295385	Alphaproteobacteria	Rhodobacteraceae	*Roseovarius *	80
DG1506	RCC 1200	KC295386	Alphaproteobacteria	Sphingomonadaceae	*Sphingopyxis *	55
DG1507	RCC 1200	KC295387	Gammaproteobacteria	Piscirickettsiaceae	*Methylophaga *	100
DG1510	RCC 1200	KC295388	Flavobacteria	Cryomorphaceae	*Wandonia *	62
DG1511	RCC 1200	KC295389	Alphaproteobacteria	Rhodobacteraceae	*Loktanella *	68
DG1512	RCC 1200	KC295390	Gammaproteobacteria	Oleiphilaceae	*Oleiphilus *	61
DG1513	RCC 1200	KC295391	Alphaproteobacteria	Hyphomonadaceae	*Hyphomonas *	65
DG1597	RCC 1200	EF140753	Gammaproteobacteria	Alteromonadaceae	*Marinobacter *	100
DG1599	RCC 1200	EU052764	Alphaproteobacteria	Rhodobacteraceae	*Maritimibacter *	59

DG1554	RCC 1203	KM279011	Alphaproteobacteria	Rhodobacteraceae	*Marivita *	100
DG1555	RCC 1203	KM279012	Alphaproteobacteria	Phyllobacteriaceae	*Hoeflea *	100
DG1556	RCC 1203	EU732746	Gammaproteobacteria	Alteromonadaceae	*Marinobacter *	100
DG1557	RCC 1203	KM279013	Alphaproteobacteria	Rhodobacteraceae	*Loktanella *	74
DG1558	RCC 1203	KM279014	Alphaproteobacteria	Rhodobacteraceae	*Roseovarius *	55
DG1561	RCC 1203	KM279015	Gammaproteobacteria	Alteromonadaceae	*Aestuariibacter *	71
DG1562	RCC 1203	KM279016	Alphaproteobacteria	Rhodobacteraceae	*Roseovarius *	62
DG1563	RCC 1203	KM279017	Alphaproteobacteria	Rhodospirillaceae	*Magnetospira *	64
DG1564	RCC 1203	KM279018	Alphaproteobacteria	Hyphomonadaceae	*Hyphomonas *	100
DG1565	RCC 1203	KM279019	Gammaproteobacteria	Oceanospirillaceae	*Oceaniserpentilla *	16
DG1566	RCC 1203	KM279020	Alphaproteobacteria	Rhodobacteraceae	*Roseovarius *	46
DG1567	RCC 1203	KM279021	Alphaproteobacteria	Rhodobacteraceae	*Roseovarius *	100
DG1568	RCC 1203	KM279022	Cytophagia	Flammeovirgaceae	*Marinoscillum *	100
DG1569	RCC 1203	KM279023	Alphaproteobacteria	Rhodobacteraceae	*Loktanella *	64
DG1570	RCC 1203	KM279024	Alphaproteobacteria	Rhodobacteraceae	*Roseovarius *	100
DG1572	RCC 1203	KM279025	Alphaproteobacteria	Rhodobacteraceae	*Sulfitobacter *	57
DG1575	RCC 1203	KM279026	Gammaproteobacteria	Gammaproteobacteria	*Porticoccus *	98
DG1576	RCC 1203	KM279027	Cytophagia	Flammeovirgaceae	*Marinoscillum *	100
DG1579	RCC 1203	KM279028	Flavobacteriia	Cryomorphaceae	*Brumimicrobium *	54
DG1582	RCC 1203	KM279029	Betaproteobacteria	Burkholderiaceae	*Limnobacter *	100

^1^Taxonomic assignment was determined using the RDP II naive Bayesian classifier [15]; Conf.: Bayesian confidence for the rank of genera only is shown.

**Table 4:** Biogeography of rare bacterial taxa in the marine environment.

Project^1^	Acidobacteria	Acidimicrobiales	*Schlegelella *	*Thermomonas *
ICoMM surface seawater (≤50 m)				
CAM	0.006	0.215	0	0.009
AWP	0.010	0.327	0	0
AOT	0.005	3.248	0	<0.001
ABR	0.001	1.435	0	0
LCR	0.038	2.696	0.004	0.008
PML	0.054	0.726	0.001	<0.001

ICoMM coral, sponge, and microbial mats				
CCB	1.368	1.065	0	0
CMM	0.955	6.806	0.001	0.002
SPO	7.718	5.318	0	0

^1^Average percentage abundance identified from each ICoMM project, normalised for sequencing effort. Data was compiled from publically accessible data at ICoMM Marine Microbes Database (http://vamps.mbl.edu/). CAM: census of Antarctic marine life [16]; AWP: Azores water profile; AOT: Atlantic Ocean transect; ABR: active but rare (Nth and Sth Pacific) [17]; LCR: latitudinal gradient from South Atlantic to the Caribbean [18]; PML, English Channel L4, UK [19]; CCB: microbial diversity in Caribbean coral species; CMM: coastal microbial mats; SPO: marine sponge-associated bacteria [20].
